# Transcriptional Regulation Mechanisms in Adaptively Evolved *Pichia kudriavzevii* Under Acetic Acid Stress

**DOI:** 10.3390/jof11030177

**Published:** 2025-02-22

**Authors:** Sureeporn Dolpatcha, Huynh Xuan Phong, Sudarat Thanonkeo, Preekamol Klanrit, Nongluck Boonchot, Mamoru Yamada, Pornthap Thanonkeo

**Affiliations:** 1 Department of Biotechnology, Faculty of Technology, Khon Kaen University, Khon Kaen 40002, Thailand; sureeporndo@kkumail.com (S.D.); kpreek@kku.ac.th (P.K.); nongke@kku.ac.th (N.B.); 2 Department of Microbial Biotechnology, Institute of Food and Biotechnology, Can Tho University, Can Tho 900000, Vietnam; hxphong@ctu.edu.vn; 3 Walai Rukhavej Botanical Research Institute, Mahasarakham University, Maha Sarakham 44150, Thailand; sudarat.t@msu.ac.th; 4 Fermentation Research Center for Value Added Agricultural Products (FerVAAPs), Khon Kaen University, Khon Kaen 40002, Thailand; 5 Department of Biological Chemistry, Faculty of Agriculture, Yamaguchi University, Yamaguchi 753-8515, Japan; m-yamada@yamaguchi-u.ac.jp; 6 Research Center for Thermotolerant Microbial Resources, Yamaguchi University, Yamaguchi 753-8515, Japan

**Keywords:** ethanol production, lignocellulosic biomass, real-time PCR, thermotolerant yeast

## Abstract

Acetic acid, a common weak acid in industrial fermentation processes, occurs naturally in lignocellulosic hydrolysates and is a byproduct of microbial metabolism. As a significant environmental stressor, it triggers the expression of multiple genes involved in various cellular responses, including biological processes, cellular components, and molecular functions. Using the acid-tolerant strain *Pichia kudriavzevii* PkAC-9, developed through adaptive laboratory evolution under acetic acid stress, we conducted a transcriptional analysis of 70 stress response-associated genes. RT-qPCR analysis revealed significant upregulation of several genes compared with the wild-type strain under acetic acid stress conditions. The most dramatic changes occurred in genes encoding key metabolic enzymes and stress response proteins associated with the TCA cycle (Fum: 18.6-fold, Aco: 17.1-fold, Oxo: 9.0-fold), carbon and energy metabolism (Tdh2: 28.0-fold, Erg2: 2.0-fold), electron transport chain (Gst: 10.6-fold), molecular chaperones (Hsp104: 26.9-fold, Hsp70: 13.0-fold, Sgt2: 10.0-fold), and transcriptional activators. Our findings indicate that the enhanced acetic acid tolerance of *P. kudriavzevii* PkAC-9 primarily depends on the coordinated upregulation of genes involved in energy metabolism, cellular detoxification mechanisms, and protein quality control systems through heat shock and transcriptional activator proteins.

## 1. Introduction

During lignocellulosic biomass processing, acetic acid accumulates from hydrolysate preparation, reaching concentrations of 1–15 g/L based on feedstock type and pretreatment conditions [1,2], and from microbial metabolism during fermentation [3,4]. This weak acid exhibits inhibitory effects on various ethanologenic microorganisms, including *Saccharomyces cerevisiae* [5,6,7], *Pichia kudriavzevii* [8,9,10,11], *P. pastoris* [12], *Kluyveromyces marxianus* [13], and *Zymomonas mobilis* [14]. At concentrations exceeding 5 g/L, acetic acid significantly impairs the microbial growth, metabolism, and fermentation efficiency, potentially leading to cell death. When present in cells, acetic acid disrupts cellular functions by penetrating the cell membrane and causing damage to vital intracellular components, such as DNA, RNA, and proteins [15,16]. To address these challenges in the fermentation processes, the development of microbial strains with enhanced acetic acid tolerance has become a key research focus.

Microbial cells employ multiple complex mechanisms to respond to acetic acid stress and operate at various cellular levels. At the membrane level, cells adapt by modifying their membrane components, reducing the membrane channel size, generating chemiosmotic gradients, and increasing the expression of proton efflux pumps [6,17,18]. To regulate intracellular pH, cells actively consume accumulated protons while simultaneously reducing proton influx [19,20]. Cells also activate crucial molecular protection mechanisms, including the activation of chaperone proteins to prevent protein denaturation and the upregulation of DNA repair systems to maintain nucleic acid integrity [5,21,22]. Additionally, cells undergo significant metabolic adjustments, enhancing their glycolytic pathway activity, increasing amino acid metabolism, and increasing RNA synthesis and processing [23]. Recent transcriptomic analyses have provided deeper insights into these stress responses, revealing the upregulation of numerous genes associated with various cellular functions. These genetic responses have been particularly well documented in yeasts, such as *S. cerevisiae* and *P. kudriavzevii*, where genes involved in biological processes, cellular components, and molecular functions are significantly activated under acetic acid stress conditions [10,24,25].

*P. kudriavzevii* has emerged as a promising non-conventional yeast for industrial applications, particularly in bioethanol and other biomolecule production. This versatile microorganism has been isolated from diverse ecological niches, including soil, fruits, and various fermented foods and beverages [8,26,27,28]. It stands out as an industrial microorganism because of its exceptional ability to withstand multiple environmental stresses commonly encountered in industrial processes, such as high temperature, elevated ethanol and sugar concentrations, and various lignocellulosic inhibitors [27,29].

In our previous study, we successfully isolated a thermotolerant strain of *P. kudriavzevii* from soil samples from the Mekong Delta region of Vietnam. This strain demonstrated notable resilience to high temperatures and high concentrations of both ethanol and acetic acid [8]. However, we observed that the ethanol fermentation efficiency was suboptimal under acetic acid stress conditions, which prompted us to pursue strain improvement strategies. Through adaptive laboratory evolution (ALE), we developed an enhanced strain designated as *P. kudriavzevii* PkAC-9. This evolved strain exhibited remarkable multi-tolerance characteristics, showing improved resistance to various industrial stressors, including heat, ethanol, osmotic pressure, and multiple inhibitory compounds such as acetic acid, formic acid, furfural, 5-(hydroxymethyl) furfural (5-HMF), and vanillin. Notably, PkAC-9 demonstrated superior ethanol fermentation efficiency under acetic acid stress compared with its parent strain [11]. To understand the molecular basis of these improved stress tolerance characteristics, particularly acetic acid resistance, we conducted a detailed transcriptional analysis using RT-qPCR. The insights gained from this analysis not only enhance our understanding of stress response mechanisms but also provide valuable guidance for the future strain development and optimization of industrial fermentation processes.

## 2. Materials and Methods

### 2.1. Chemicals and Culture Media

The main chemicals used in this study were obtained from various manufacturers. Yeast extract, malt extract, and peptone (bacteriological grade) were obtained from TM Media (Titan Biotech Ltd., Delhi, India). Glucose (D(+)-glucose 1-hydrate) were purchased from KemAusTM (New South Wales, Australia), while glacial acetic acid was acquired from Sigma-Aldrich (St. Louis, MI, USA). Isopropanol and absolute ethanol (HPLC grade) were procured from Sigma-Aldrich.

The YM liquid medium contained the following components: 3 g/L yeast extract, 3 g/L malt extract, 5 g/L peptone, and 10 g/L glucose. To prepare the solid medium, 15 g/L agar was added to the formulation. Before use, all the culture media were sterilized by autoclaving at 121 °C for 15 min.

### 2.2. Yeast Strains and Culture Conditions

In this study, two *P. kudriavzevii* strains were used: CM4.2, a thermotolerant yeast isolated from soil [8], and PkAC-9, a strain evolved through adaptive laboratory evolution (ALE) under acetic acid stress [11]. The ALE protocol was adapted from Samappito et al. [14] and consisted of three sequential steps with increasing acetic acid concentrations. For each step, cultures were maintained in 50 mL YM broth at 35 °C with shaking at 150 rpm. The initial inoculum was standardized to 1 × 10^6^ cells/mL. The evolution endpoint for each cycle was determined by cell density reaching 1 × 10^8^ cells/mL, indicating stable growth under selective pressure. In the first step, cells were cultured in a medium containing 7 g/L acetic acid with 48 h cultivation cycles. After each cycle, cells were transferred to a fresh medium maintaining the same inoculum size. This process continued for 24 cycles. The second step employed 8 g/L acetic acid with 72 h cultivation cycles for 45 cycles. Cells showing stable growth from the first step served as the starting culture. In the final step, the acetic acid concentration in the medium was increased to 9 g/L, and the process continued for 53 cycles using the same cultivation parameters as the second step. The resulting evolved strain, PkAC-9, was obtained and used in this study [11].

The yeast strains were cultivated in 100 mL of YM liquid medium in a controlled incubator shaker (JSR, Gongju, Republic of Korea) at 35 °C and 150 rpm. After 16 h of incubation, the cells were transferred to a fresh YM liquid medium (100 mL) at an initial concentration of 10^5^ cells/mL. The cultures were then incubated for an additional 12 h under the same conditions (35 °C and 150 rpm) to generate active cells for use as starter cultures in subsequent experiments.

### 2.3. Ethanol Production Under Acetic Acid Stress

The ethanol production capability of the wild-type and evolved *P. kudriavzevii* strains was evaluated using YM medium containing 160 g/L glucose and varying concentrations of acetic acid, following the methodology of Dolpatcha et al. [11]. Batch fermentation was conducted in 250 mL Erlenmeyer flasks containing 100 mL YM medium supplemented with 7, 8, or 9 g/L acetic acid, with an unsupplemented medium serving as a control. Each flask was inoculated with a starter culture to achieve an initial cell concentration of approximately 1 × 10^7^ cells/mL and was incubated in a controlled shaker at 35 °C and 150 rpm. Throughout the fermentation process, samples were randomly collected at predetermined intervals, and ethanol concentrations were measured using gas chromatography (GC).

### 2.4. RT-qPCR for Gene Expression Analysis Under Acetic Acid Stress

#### 2.4.1. RNA Isolation

Both *P. kudriavzevii* strains, CM4.2 and PkAC-9, were cultivated in YM liquid medium under control (0 g/L acetic acid) and stress (9 g/L acetic acid) conditions at 35 °C and 150 rpm in a controlled incubator shaker. Following Dolpatcha et al. [11], cultures were grown until they reached the mid-exponential phase, which occurred at 8 h under control conditions and at 40 h under acid-stress conditions. The cells were then harvested by centrifugation at 5000 rpm at 4 °C for 10 min and washed twice with sterile deionized water. RNA was isolated from the cell pellets using TRIzol^®^ reagent (Thermo Fisher Scientific, Waltham, MA, USA) according to the manufacturer’s protocol. The isolated RNA was quantified and assessed for quality using a BioDrop μLite spectrophotometer (Denville Scientific Inc., South Plainfield, NJ, USA) prior to the gene expression analysis.

#### 2.4.2. RT-qPCR Analysis

Gene expression analysis was performed using the Applied Biosystems^TM^ QuantStudio^TM^ 5 Real-Time PCR system with MeltDoctor^TM^ HRM Master Mix (Applied Biosystems, Waltham, MA, USA), following the manufacturer’s protocols for reaction mixture preparation and thermal cycling conditions. A comprehensive set of 70 genes, selected through a literature review, was investigated, with the actin gene serving as an internal control [10,24,25]. Primer sets used in this study are listed in Supplementary Material Table S1. Gene expression levels were quantified in triplicate using the comparative critical threshold (2^−ΔΔCT^) method as described by [30].

### 2.5. Analytical Methods and Statistical Analysis

Ethanol concentration (*P*, g/L) was measured using a gas chromatograph (GC-14B, Shimadzu, Kyoto, Japan) equipped with a polyethylene glycol (PEG-20M) packed column and a flame ionization detector (FID). Isopropanol was used as the internal standard [31]. Volumetric ethanol productivity (*Qp*, g/L·h) and ethanol yield (*Yp*/*s*, g/g) were calculated according to the method described by Nuanpeng et al. [32].

The ethanol fermentation experiments were conducted in duplicate with two replicates each, and the results are expressed as mean ± standard deviation (SD). Statistical analyses were performed using IBM SPSS Statistics version 28 (IBM Corporation, Armonk, NY, USA). The mean differences between treatments were analyzed using Duncan’s Multiple Range Test (DMRT) at a 5% probability level (*p* ≤ 0.05).

## 3. Results and Discussion

*P. kudriavzevii* PkAC-9 was developed through ALE under increasing acetic acid concentrations (7–9 g/L) at a selective pressure [11]. The evolution experiment, conducted over 122 cultivation cycles, resulted in stable growth with cell densities consistently reaching 10^8^ cells/mL under acetic acid stress. This adaptation occurred through three sequential phases: 24 cycles at 7 g/L, 45 cycles at 8 g/L, and 53 cycles at 9 g/L acetic acid, demonstrating the strain’s acquired tolerance to progressively higher concentrations of acetic acid. In this study, we investigated both the ethanol production capability of PkAC-9 and the molecular mechanisms underlying its enhanced acetic acid tolerance. Our findings are presented as follows:

### 3.1. Ethanol Production Under Acetic Acid Stress

Acetic acid is one of the most abundant weak acids generated during the pretreatment of lignocellulosic materials, particularly under severe conditions, such as high-acid or thermal treatment [2,11]. The concentration of acetic acid in lignocellulosic hydrolysates can reach up to 15 g/L, varying with the feedstock type and pretreatment conditions. High concentrations of this weak acid have been shown to inhibit growth and impair fermentation activity in various ethanologenic microorganisms, including *K. marxianus* [13], *P. kudriavzevii* [9,11], and *Z. mobilis* [14,33].

Dolpatcha et al. [11] demonstrated that high concentrations of acetic acid significantly reduced the specific growth rate of both wild-type and evolved strains of *P. kudriavzevii*. However, its effect on ethanol fermentation efficiency remains unexplored. Therefore, this study investigated the effect of acetic acid on ethanol production by wild-type and evolved *P. kudriavzevii* strains; the results are summarized in Table 1.

The results revealed that the wild-type *P. kudriavzevii* strain was more sensitive to acetic acid than the evolved strain. While the ethanol production performance of the wild-type strain significantly decreased with increasing acetic acid concentrations, the evolved strain maintained remarkably higher ethanol concentration, volumetric productivity, and yield, particularly under acetic acid stress. High concentrations of acetic acid extended the fermentation time by inhibiting the fermentation ability of both the wild-type and evolved strains. This inhibition resulted from the negative effects of acetic acid on cellular membrane structure and protein stability. Previous studies have shown that high acetic acid concentrations inhibit transmembrane proton transport, disrupt the cellular membrane structure, and denature cellular proteins [34,35].

Notably, the evolved strain, based on ethanol production efficiency measured by ethanol concentration and yield, showed greater resistance to acetic acid stress. This enhanced tolerance suggests the development of acetic acid resistance mechanisms during the long-term adaptation of this strain to acetic acid stress [11].

### 3.2. RT-qPCR Analysis of Gene Expression Under Acetic Acid Stress

Differences in ethanol production efficiency between wild-type and evolved *P. kudriavzevii* strains suggest distinct acid tolerance mechanisms. Previous transcriptional analyses have revealed the key pathways involved in acid tolerance. Under hydrochloric acid stress, Akita and Matsushika [24] identified 14 enriched pathways in *P. kudriavzevii* strains NBRC1279 and NBRC1664, spanning biological processes (including basal transcription factors, the biosynthesis of amino acids, the biosynthesis of secondary metabolites, the tricarboxylic acid cycle (TCA cycle), glycerophospholipid metabolism, longevity regulating pathway-multiple species, mitophagy-yeast, phagosome, proteasome, RNA degradation, and RNA transport), cellular components (including the MAPK signaling pathway–yeast and protein processing in the endoplasmic reticulum), and molecular functions (specifically oxidative phosphorylation). Similarly, Wang et al. [10] found that differentially expressed genes (DEGs) in *P. kudriavzevii* Y2 under acetic acid stress were primarily enriched in biological processes (ribonucleoprotein complex biogenesis, ribosome biogenesis, and oxidation-reduction processes), cellular components (pre-ribosome and membrane processes), and molecular functions (oxidoreductase activity). These findings guided our selection of key genes for RT-qPCR analysis under both normal and acetic acid stress conditions.

The mitogen-activated protein kinase (MAPK) signaling pathway transmits signals from cell surface receptors to nuclear DNA, mediating cellular responses to external stimuli, such as stress, growth factors, and cytokines. This conserved pathway regulates fundamental processes, including cell proliferation, differentiation, survival, and stress responses [36,37]. In yeasts such as *S. cerevisiae* and *P. kudriavzevii*, MAPK signaling, particularly the high-osmolarity glycerol (HOG) pathway, is crucial for acid stress tolerance [24].

**Figure 1:**
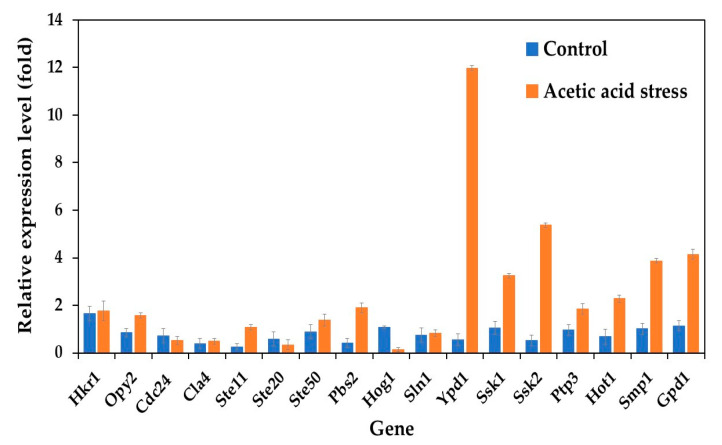
Differential expression of genes related to the HOG pathway in *P. kudriavzevii* PkAC-9 compared with the wild-type strain under normal and acetic acid stress. The relative expression levels were calculated using the comparative critical threshold (2^−ΔΔCT^) method, with the wild-type strain serving as the reference strain (control strain) and actin as the internal control gene. Bars represent the fold change in gene expression in the evolved strain (PkAC-9) relative to the wild-type strain under two conditions: control (without acid stress) and acetic acid stress (9 g/L). A fold change of 1 indicates an expression equal to the wild-type strain under the respective condition.

Analysis of HOG pathway-related genes revealed distinct expression patterns between the wild-type and evolved *P. kudriavzevii* strains. Under control conditions, all studied genes were upregulated in the evolved strain. Upon acetic acid stress exposure, most genes were highly upregulated, except for Cdc24, Ste20, and Hog1, which were downregulated compared to control conditions (Figure 1). Notably, seven genes, including Ypd1, Ssk1, Ssk2, Ptp3, Hot1, Smp1, and Gpd1, exhibited remarkably higher expression levels under acetic acid stress, with Ypd1 showing an approximately 21-fold increase. These findings suggested that the enhanced acetic acid tolerance of the evolved strain *P. kudriavzevii* PkAC-9 likely results from the elevated expression of these HOG pathway genes, which promote glycerol synthesis to increase internal osmotic pressure under stress conditions. Glycerol is an important metabolite of alcoholic fermentation and is involved in maintaining the intracellular osmotic balance required for growth under acid stress. The results obtained in the current study are consistent with previous observations by Akita and Matsushika [24], who reported the expression of HOG pathway genes in *P. kudriavzevii* NBRC1279 and NBRC1664 under acid stress.

Studies have demonstrated that budding yeast *S. cerevisiae* employs multiple regulatory mechanisms to combat acetic acid stress, with transcriptional activation being a crucial defense strategy [18]. Recent investigations have identified and characterized numerous genes encoding transcriptional activators in both *S. cerevisiae* and *P. kudriavzevii*, highlighting their significance in stress response [7,10,24,25].

**Figure 2:**
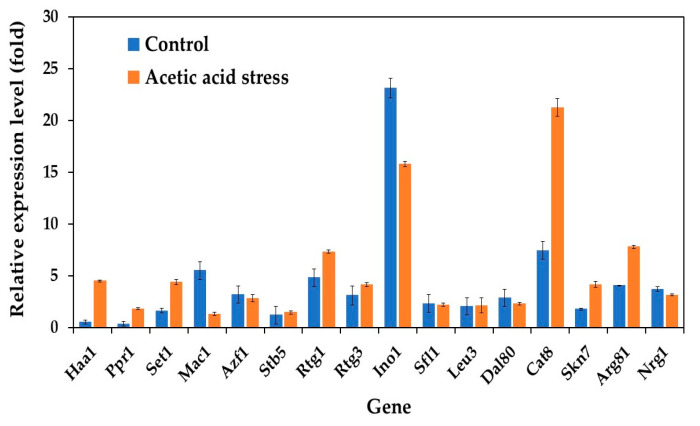
Differential expression of genes related to transcriptional activators in *P. kudriavzevii* PkAC-9 compared to the wild-type strain under normal and acetic acid stress. The relative expression levels were calculated using the comparative critical threshold (2^−ΔΔCT^) method, with the wild-type strain serving as the reference strain (control strain) and actin as the internal control gene. Bars represent the fold change in gene expression in the evolved strain (PkAC-9) relative to the wild-type strain under two conditions: control (without acid stress) and acetic acid stress (9 g/L). A fold change of 1 indicates an expression equal to the wild-type strain under the respective condition.

In our study, we examined the expression profiles of 16 key transcriptional activator genes (Haa1, Ppr1, Set1, Mac1, Azf1, Stb5, Rtg1, Rtg3, Ino1, Sfl1, Leu3, Dal80, Cat8, Skn7, Arg81, and Nrg1) in both wild-type and evolved strains of *P. kudriavzevii*. Under normal conditions without acid stress, the evolved strain exhibited distinct gene expression patterns compared to those of the wild-type strain. Notably, while most genes maintained low basal expression, five genes, including Mac1, Azf1, Ino1, Dal80, and Nrg1, showed expression levels that surpassed those observed under acetic acid stress conditions (Figure 2).

While initially unexpected, this expression pattern aligns with known regulatory mechanisms in yeast stress responses. These transcription factors often maintain higher baseline expression under normal conditions as part of the cell’s stress preparedness system. Their activity is primarily regulated through post-translational modifications and protein relocalization rather than increased transcription under stress conditions. For instance, Mac1, involved in metal ion homeostasis, undergoes conformational changes in response to stress rather than requiring increased gene expression. Azf1, which regulates glucose-responsive genes, maintains elevated baseline expression to enable rapid metabolic adaptation. Ino1, involved in inositol biosynthesis, is primarily regulated through phosphorylation. Dal80 and Nrg1, known negative regulators of stress response genes, are actually expected to show reduced expression under stress conditions, as their downregulation helps activate stress response pathways [38,39,40].

These findings contrast with those of previous studies on acid-tolerant *Zygosaccharomyces bailii* and *P. kudriavzevii*, where transcription activator genes, particularly Azf1 and Mac1 (a copper ion-sensing transcription protein), were upregulated in response to acetic acid or low-pH stress [25,41]. This regulatory strategy in our evolved strain suggests an alternative mechanism where cells maintain a ready pool of transcription factors that can be rapidly activated through post-translational modifications when needed, rather than relying on new protein synthesis, which would be more energetically costly and slower during stress conditions. These findings indicate that acid tolerance mechanisms through transcriptional regulation are species-specific stress response mechanisms.

The evolved strain exhibited significant transcriptional remodeling upon exposure to acetic acid. Three transcriptional activators showed particularly strong upregulation: Haa1 displayed an 8.4-fold increase, whereas Ppr1 and Cat8 showed 5.2-fold and 2.9-fold increases, respectively, compared to unstressed conditions. Additionally, genes involved in the mitochondrial retrograde (RTG) pathway (Rtg1 and Rtg3) and histone methyltransferase (Set1), known for their roles in the acetic acid stress response [25,42], were also upregulated in the evolved strain under acid stress. This enhanced transcriptional response in the evolved strain suggests the development of a more robust stress-adaptation mechanism through laboratory evolution. These findings extend our understanding beyond previous research in *S. cerevisiae*, where Haa1 has been established as a central regulator of acetic acid stress response. In *S. cerevisiae*, Haa1’s regulatory network includes crucial downstream targets such as Hrk1, which encodes a plasma membrane H+-ATPase essential for cellular pH homeostasis, and the membrane transporter genes Tpo2 and Tpo3, which facilitate acetic acid detoxification [18,43,44]. The remarkable upregulation of Haa1 (8.4-fold) observed specifically in our evolved *P. kudriavzevii* strain not only suggests the conservation of this stress response mechanism across yeast species but also indicates the potential enhancement of this pathway through adaptive evolution. This evolved response likely represents a key mechanism underlying the improved acetic acid tolerance of this strain.

**Figure 3:**
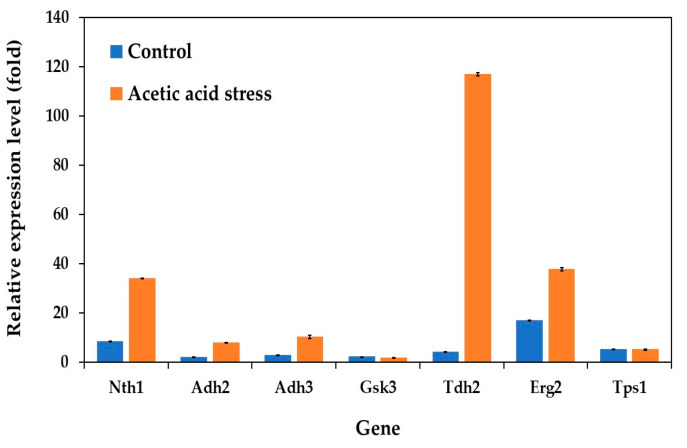
Differential expression of genes related to carbon and energy metabolism in *P. kudriavzevii* PkAC-9 compared to the wild-type strain under normal and acetic acid stress. The relative expression levels were calculated using the comparative critical threshold (2^−ΔΔCT^) method, with the wild-type strain serving as the reference strain (control strain) and actin as the internal control gene. Bars represent the fold change in gene expression in the evolved strain (PkAC-9) relative to the wild-type strain under two conditions: control (without acid stress) and acetic acid stress (9 g/L). A fold change of 1 indicates an expression equal to the wild-type strain under the respective condition.

Analysis of the gene expression patterns related to carbon and energy metabolism revealed distinct differences between the evolved and wild-type strains under control conditions. The evolved strain demonstrated consistently lower expression levels of several key metabolic genes, including those encoding alcohol dehydrogenases (Adh2 and Adh3), glycogen synthase kinase (Gsk3), glyceraldehyde-3-phosphate dehydrogenase or GAPDH (Tdh2), and trehalose-6-phosphate synthase 1 (Tps1). Notably, Erg2, which encodes sterol C-8 isomerase in the ergosterol biosynthesis pathway, exhibited the highest expression levels among the analyzed genes (Figure 3). This elevated expression was observed both under control conditions and under acid stress, where the evolved strain showed significant upregulation compared to the wild type. The consistent upregulation of Erg2 suggests a crucial role of ergosterol in the growth and development of the evolved strain under both normal and stress conditions.

In *S. cerevisiae*, ergosterol serves as the primary fungal sterol and is essential for cellular membrane integrity and mitochondrial DNA maintenance [45,46]. Its importance in stress response and cellular development is further emphasized by studies showing that the disruption of ergosterol biosynthesis results in mitochondrial DNA loss, demonstrating the interconnected relationship between mitochondrial function and ergosterol metabolism [46].

Under acetic acid stress, the evolved strain exhibited significant gene expression changes compared to the control conditions. Most notably, Tdh2 showed an exceptional 28-fold increase, substantially higher than other upregulated genes such as Nth1 (4-fold) and Erg2 (2-fold). The dramatic upregulation of Tdh2, which encodes GAPDH, aligns with its known role as a multifunctional protein. Beyond its primary function in glycolysis, GAPDH participates in diverse cellular processes, including genome stability, DNA repair, tRNA export, membrane fusion and transport, cytoskeletal dynamics, and cell death processes [47,48,49,50,51,52]. Recent studies have particularly highlighted GAPDH’s crucial role in oxidative stress response and DNA damage repair [53]. Its dual localization in both the yeast cell surface and cytosol is essential for maintaining cellular membrane integrity and protein stability under severe stress conditions [34,35].

While the moderate upregulation of Nth1 (neutral trehalase) and Erg2 (C-8 sterol isomerase) suggests their supporting roles in stress adaptation, their response was notably less pronounced compared to Tdh2. Interestingly, the role of these genes in stress responses varies significantly among different yeast species. For instance, while Tdh2 is strongly upregulated in *P. kudriavzevii* under acetic acid stress, it is actually downregulated in *S. cerevisiae* under similar conditions [12], highlighting a distinct species-specific stress response mechanism. In other yeasts like *Schizosaccharomyces pombe*, GAPDH has been reported to play crucial roles in oxidative stress responses and cellular protection through different regulatory patterns [54]. Additionally, the unchanged expression of Gsk3 and Tps1 under both control and stress conditions (Figure 3) indicates that glycogen and trehalose metabolism may play minimal roles in the response of *P. kudriavzevii* to acetic acid stress, unlike in some other yeast species.

Heat shock proteins (HSPs) function as molecular chaperones and play crucial roles in cellular stress response. These proteins assist in proper folding, help refold denatured proteins, and prevent protein aggregation. Additionally, some HSPs act as proteases to degrade denatured proteins and are involved in maintaining membrane integrity and cellular homeostasis [55].

**Figure 4:**
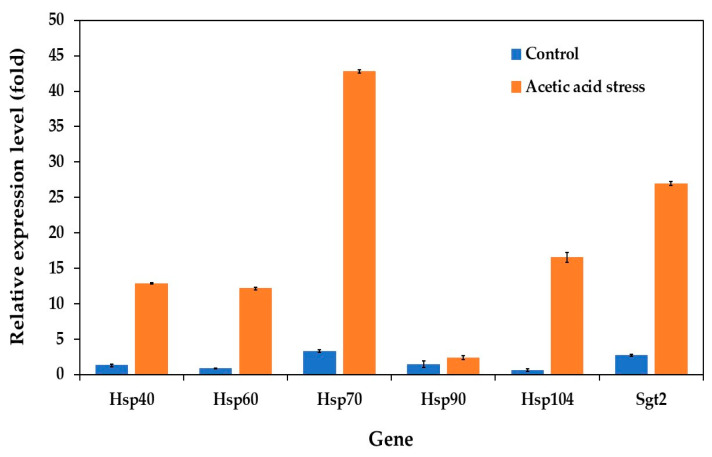
Differential expression of genes related to heat shock proteins in *P. kudriavzevii* PkAC-9 compared to the wild-type strain under normal and acetic acid stress. The relative expression levels were calculated using the comparative critical threshold (2^−ΔΔCT^) method, with the wild-type strain serving as the reference strain (control strain) and actin as the internal control gene. Bars represent the fold change in gene expression in the evolved strain (PkAC-9) relative to the wild-type strain under two conditions: control (without acid stress) and acetic acid stress (9 g/L). A fold change of 1 indicates an expression equal to the wild-type strain under the respective condition.

In the *P. kudriavzevii* evolved strain, the expression patterns of the HSP genes varied significantly between normal and stress conditions. Under normal growth conditions, HSP genes, including Hsp40, Hsp60, Hsp70, Hsp90, Hsp104, and the co-chaperone Sgt2, showed relatively low expression levels. However, upon exposure to acetic acid stress, all genes except Hsp90 were significantly upregulated, with Hsp70 showing the highest expression, followed by Sgt2 and Hsp104 (Figure 4). These expression patterns indicate a stronger stress response in the evolved strain than in the wild-type strain.

The substantial upregulation of heat shock protein genes, particularly HSP70, plays a crucial role in cellular adaptation to acid stress through multiple mechanisms. In acidic environments, protein structures are compromised due to disrupted hydrogen bonds and electrostatic interactions. HSP70 specifically recognizes and binds to exposed hydrophobic regions of destabilized proteins, preventing their aggregation and facilitating proper refolding. This chaperone function becomes especially critical as acetic acid triggers oxidative stress pathways, leading to increased production of reactive oxygen species (ROS) and subsequent protein carbonylation. Under these conditions, HSP70 works synergistically with other stress response proteins to either assist in protein refolding or target severely damaged proteins for degradation, while simultaneously supporting the function of antioxidant enzymes such as catalase and superoxide dismutase [56,57,58].

Beyond protein quality control, HSP70’s protective role extends to maintaining membrane stability, which is particularly crucial during acid stress when decreased pH disrupts membrane lipid organization and protein–lipid interactions. HSP70 contributes to membrane integrity through three key mechanisms: (1) stabilizing membrane-associated proteins, (2) facilitating proper protein trafficking, and (3) supporting the assembly and disassembly of protein complexes essential for stress response [58,59]. This multi-faceted protective function explains the significant upregulation of HSP70 observed in our study, as it serves as a central coordinator of both protein homeostasis and membrane stability under acid-stress conditions.

Acetic acid stress negatively affects the plasma membrane and cell wall structure of yeasts. Previous studies have demonstrated that acetic acid accumulation alters the molecular composition and physical properties of the cell envelope, resulting in decreased membrane permeability [6,60,61]. Transcriptomic analyses of *S. cerevisiae* have revealed that genes encoding proteins involved in cell wall polysaccharide synthesis and remodeling are upregulated in response to acetic acid stress [6,18,62].

**Figure 5:**
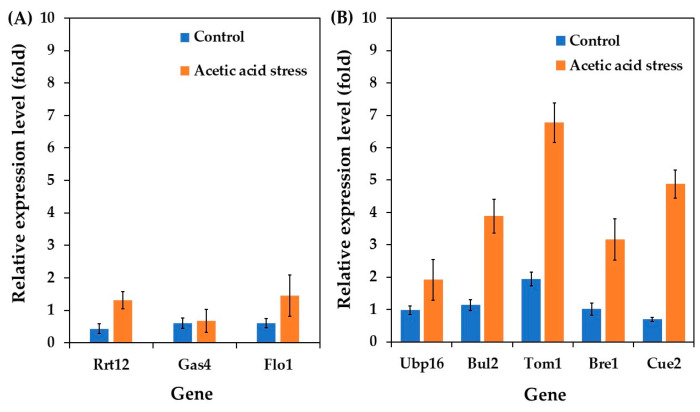
Differential expression of genes related to stress membrane biogenesis (**A**) and ubiquitin-proteasome (**B**) in *P. kudriavzevii* PkAC-9 compared with the wild-type strain under normal and acetic acid conditions. The relative expression levels were calculated using the comparative critical threshold (2^−ΔΔCT^) method, with the wild-type strain serving as the reference strain (control strain) and actin as the internal control gene. Bars represent the fold change in gene expression in the evolved strain (PkAC-9) relative to the wild-type strain under two conditions: control (without acid stress) and acetic acid stress (9 g/L). A fold change of 1 indicates an expression equal to the wild-type strain under the respective condition.

Motivated by these findings, we investigated the expression of cell wall-related genes in the acetic acid-tolerant yeast *P. kudriavzevii* PkAC-9, focusing on three key genes: Rrt12 (a protein involved in ascospore wall assembly), Gas4 (β-1,3-glucanosyltransferase involved in β-glucan elongation and branching), and Flo1 (flocculin involved in flocculation). As shown in Figure 5A, all investigated genes were upregulated under control conditions compared to the wild type. Notably, the expression of Rrt12 and Flo1 increased remarkably under acidic stress conditions, with Gas4 showing a slight increase.

The upregulation of Gas4 and Flo1 aligns with previous research on *S. cerevisiae*. Ribeiro et al. [6] reported increased mRNA levels of the Gas gene during acetic acid stress, contributing to a stiffer and more robust cell wall that limits the futile cycle of toxic acid re-entry. Similarly, Du et al. [63] demonstrated that Flo1 overexpression enhances *S. cerevisiae* fermentation performance under acetic acid stress. Further studies by Li et al. [64] and Cheng et al. [65] have highlighted that flocculating yeasts exhibit better stress resistance than non-flocculating strains. Based on the observed upregulation of Gas4 and Flo1, we propose that the ability of *P. kudriavzevii* to withstand acetic acid stress may be attributed to improved cell wall composition, structure, and increased flocculation activity.

Interestingly, the Rrt12 gene was upregulated in *P. kudriavzevii* under acetic acid stress, contrasting with its downregulation in *S. cerevisiae* [62]. This differential response likely reflects the distinct evolutionary adaptations between these species. In *P. kudriavzevii*, Rrt12 upregulation may contribute to its superior acid tolerance through enhanced cell wall remodeling and stress signaling pathways. The protein’s interaction with the HOG pathway in *P. kudriavzevii* differs from its role in *S. cerevisiae*, where it primarily functions in ribosome biogenesis and ascospore formation [62,66]. These species-specific differences in Rrt12 regulation and function highlight the diverse molecular strategies employed by different yeasts to cope with acid stress.

The ubiquitin system plays a crucial role in diverse cellular processes, including homeostasis, cell cycle regulation, DNA repair, apoptosis, gene expression, and stress responses [67]. Hershko and Ciechanover [68] reported that ubiquitin is essential for marking proteins for selective degradation by ligating them to protein substrates and forming multi-ubiquitin chains, which are then recognized and degraded by the 26S proteasome in an energy-dependent manner.

Several ubiquitin genes have been shown to be responsible for environmental stress responses, such as heat and salt stress [67,69,70]. In this study, we investigated the differential expression of ubiquitin genes, including Ubp16, Bul2, Tom1, Bre1, and Cue2, in acetic acid-tolerant *P. kudriavzevii* PkAC-9 strain under acetic acid stress. The findings revealed that all examined genes exhibited higher expression levels than the wild-type strain under control conditions. Interestingly, their expression was significantly upregulated in response to acetic acid stress. These results are consistent with those of previous studies on *S. cerevisiae*. For instance, Kinner and Kölling [71] reported that Ubp16 plays an essential role in ubiquitin homeostasis under various circumstances. Yashiroda et al. [69] reported that Bul proteins are critical for *S. cerevisiae* growth under stressful conditions, including high temperatures, salts, and non-fermentable carbon sources. Sasaki et al. [67] demonstrated that Tom1 is essential for *S. cerevisiae* growth at high temperatures, and gene deletion results in reduced cell growth. Zhang et al. [70] highlighted Bre1’s involvement in DNA damage response and repair, ensuring genomic structural integrity. Simms et al. [72] and Johnston et al. [73] reported that Cue2 is involved in the mRNA decay pathway and the degradation of misfolded or damaged proteins under stressful conditions.

Since acetic acid stress can cause RNA structural abnormalities and protein denaturation, the upregulation of these ubiquitin genes observed in the current study may help the *P. kudriavzevii* PkAC-9 strain resist the severe effects of stress by maintaining cellular homeostasis and protein quality control.

Previous studies have shown that both low and high acetic acid concentrations trigger the expression of electron transport chain (oxidative phosphorylation) genes in *P. kudriavzevii* [10]. To further investigate this response, we analyzed the expression levels of key genes under control and acid stress conditions in both the wild-type and evolved strains. The genes examined included NADH dehydrogenase (Nadh-de), succinate dehydrogenase (Sdh), ubiquinol-cytochrome C reductase (CyC-R), mitochondrial cytochrome C oxidase (CyC-O), proton transporting ATPase (ATPase), pyrimidodoazepine synthase (Gsh), and glutathione S-transferase (Gst).

**Figure 6:**
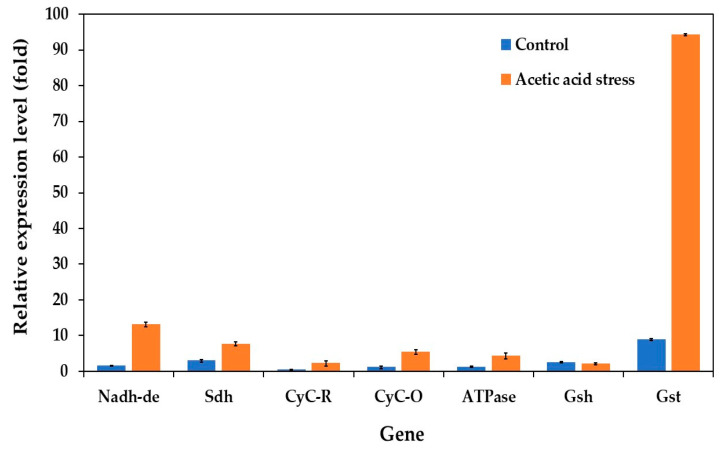
Differential expression of genes related to the electron transport chain (oxidative phosphorylation) in *P. kudriavzevii* PkAC-9 compared with the wild-type strain under normal and acetic acid stress. The relative expression levels were calculated using the comparative critical threshold (2^−ΔΔCT^) method, with the wild-type strain serving as the reference strain (control strain) and actin as the internal control gene. Bars represent the fold change in gene expression in the evolved strain (PkAC-9) relative to the wild-type strain under two conditions: control (without acid stress) and acetic acid stress (9 g/L). A fold change of 1 indicates an expression equal to the wild-type strain under the respective condition.

Under control conditions without acid stress, the evolved strain showed notably low expression levels for almost all evaluated genes (Figure 6). However, several genes, particularly Gst, Nadh-de, and Sdh, were significantly upregulated under acetic acid stress. Specifically, the evolved strain showed approximately 10.6-, 8.4-, and 2.6-fold higher expression of the Gst, Nadh-de, and Sdh genes, respectively, than the wild-type strain under acid stress. The Gsh gene expression remained relatively unchanged under these conditions. These findings align with those of the previous studies by Zhang et al. [74] and Wang et al. [10].

The upregulation of oxidative phosphorylation genes reflects increased ATP demand for cellular metabolism under stress conditions. Similar responses have also been observed in other organisms. In *Streptomyces albulus*, genes related to succinate-Q reductase and cytochrome C oxidase were significantly upregulated under acidic conditions, whereas ATP synthase genes (atpH, atpF, and atpE) were downregulated [74]. This downregulation appears to limit H+ influx into the cell, preventing intracellular acidification, and maintaining cellular homeostasis. Similarly, in *S. cerevisiae*, the major genes involved in NADH/NADPH regeneration reactions are upregulated under acidic conditions, enabling the normal biosynthesis of essential macromolecules such as amino acids, nucleic acids, and lipids [75].

The expression of genes involved in the citrate cycle (TCA cycle) was significantly upregulated under acetic acid stress, complementing previously observed changes in oxidative phosphorylation genes. This response aligns with the findings of Wang et al. [10] and Dubinkina et al. [25]. Our study evaluated the expression of key TCA cycle genes under both control and acid stress conditions in the wild-type and evolved strains. These genes include phosphoenolpyruvate carboxykinase (Phc), pyruvate decarboxylase (Pdc), malate dehydrogenase (Mad), citrate synthase (Csy), aconitate hydratase (Aco), fumarate hydratase (Fum), isocitrate dehydrogenase (Iso), succinyl-CoA synthetase (SucCoA), and 2-oxoglutarate dehydrogenase (Oxo).

**Figure 7:**
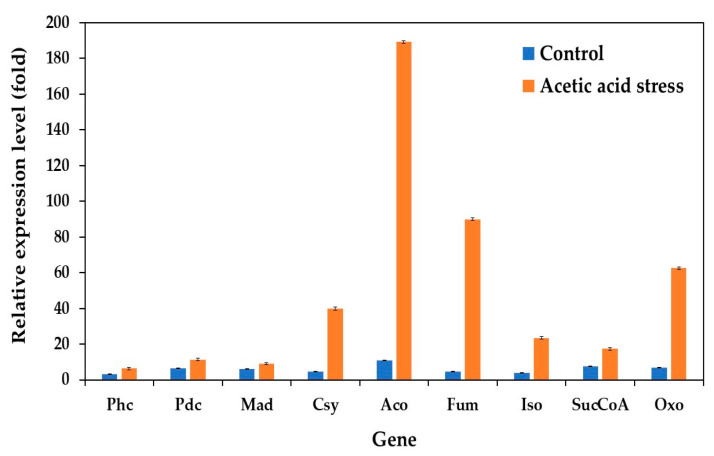
Differential expression of genes related to citrate cycle (TCA cycle) in *P. kudriavzevii* PkAC-9 compared to wild-type strain under normal and acetic acid stress. The relative expression levels were calculated using the comparative critical threshold (2^−ΔΔCT^) method, with the wild-type strain serving as the reference strain (control strain) and actin as the internal control gene. Bars represent the fold change in gene expression in the evolved strain (PkAC-9) relative to the wild-type strain under two conditions: control (without acid stress) and acetic acid stress (9 g/L). A fold change of 1 indicates an expression equal to the wild-type strain under the respective condition.

While the expression levels remained relatively low under control conditions, acid stress induced substantial upregulation of these genes. The most dramatic increases were observed in Fum (18.6-fold), Aco (17.1-fold), Oxo (9.0-fold), and Csy (8.3-fold), compared to the control conditions without acid stress (Figure 7). These results are consistent with those of previous studies by Li et al. [76], Paes et al. [12], Wang et al. [10], and Dubinkina et al. [25].

The TCA cycle generates energy through the oxidation of acetyl coenzyme A (CoA), derived from carbohydrates, proteins, and fatty acids, primarily producing adenosine triphosphate (ATP) [77]. The upregulation of TCA cycle genes under acid stress has been observed in various organisms. For instance, the upregulation of genes related to the TCA cycle and oxidoreductase activity was observed in *P. kudriavzevii* under low-pH conditions [25] as well as in *P. pastoris* upon acetic acid stress [12]. In *S. cerevisiae*, both glycolytic pathway and TCA cycle genes show increased expression under acetic acid, formic acid, and a mixture of both acids [76]. Similarly, *St. albulus* utilizes the TCA cycle as its primary pathway for energy supply and substrate oxidation under low-pH stress, with significant upregulation of citrate synthase, the α-ketoglutarate dehydrogenase complex, and succinate dehydrogenase [74]. Acetic acid-resistant *Escherichia coli* also exhibits an upregulation of key TCA cycle genes, including phosphoenolpyruvate carboxykinase, malate dehydrogenase, and isocitrate dehydrogenase, under acid stress [78]. The observed upregulation of most TCA cycle genes in the evolved *P. kudriavzevii* PkAC-9 strain suggests that enhanced TCA cycle activity serves as a crucial mechanism for generating the substantial energy required to resist acetic acid stress.

This study identified several genes that contribute to acetic acid tolerance in *P. kudriavzevii* evolved strain PkAC-9. While gene expression patterns varied under both control and acidic conditions, genes associated with the TCA cycle, carbon and energy metabolism, and electron transport chain showed the most significant upregulation under acetic acid stress. The current findings are consistent with previous reports in *S. cerevisiae* and other acid-tolerant microorganisms, as previously discussed. Based on these results, we hypothesized that the acetic acid tolerance of *P. kudriavzevii* PkAC-9 primarily depends on genes related to the TCA cycle, carbon and energy metabolism, and electron transport chain pathways.

RT-qPCR analysis can help identify potential key genes involved in the acid tolerance of *P. kudriavzevii* PkAC-9. To definitively understand their roles, further research should include direct mutagenesis of these candidate genes, particularly Aco (encoding aconitate hydratase), Tdh2 (encoding glyceraldehyde-3-phosphate dehydrogenase), Fum (encoding fumarate hydratase), oxo (encoding 2-oxoglutarate dehydrogenase), and Gst (encoding glutathione S-transferase) genes. Additionally, *P. kudriavzevii* PkAC-9 demonstrated tolerance to heat, ethanol, osmotic stress, formic acid, furfural, 5-(hydroxymethyl) furfural (5-HMF), and vanillin [11], warranting comprehensive transcriptomic analysis under these stress conditions.

## 4. Conclusions

Transcriptional analysis of the acid-tolerant *P. kudriavzevii* strain PkAC-9 revealed specific and quantifiable genetic adaptations to acetic acid stress. While gene expression remained low under control conditions, exposure to acetic acid triggered substantial and targeted transcriptional reprogramming, with several key genes showing remarkable upregulation. The most pronounced changes occurred in three critical pathways: First, central carbon metabolism showed dramatic activation, particularly in TCA cycle genes, with Fum, Aco, and Oxo experiencing 18.6-, 17.1-, and 9.0-fold increases, respectively. Second, cellular energy systems demonstrated substantial upregulation, notably through Tdh2 (28.0-fold increase) in carbon metabolism and Gst (10.6-fold increase) in the electron transport chain. Third, cellular protection mechanisms were significantly enhanced, especially through molecular chaperone systems, with Hsp104, Hsp70, and Sgt2 showing 26.9-, 13.0-, and 10.0-fold increases, respectively.

This coordinated upregulation of specific metabolic and protective pathways explains the enhanced acetic acid tolerance of the evolved strain. The magnitude of these changes, particularly in energy metabolism (up to 28.0-fold) and cellular protection systems (up to 26.9-fold), demonstrates the effectiveness of adaptive laboratory evolution in developing industrial strains. These findings provide both mechanistic insights into yeast stress tolerance and a quantitative foundation for engineering robust strains for second-generation bioethanol production from lignocellulosic feedstocks.

## Figures and Tables

**Table 1:** Ethanol production by *P. kudriavzevii* CM4.2 and PkAC-9 in YM liquid medium containing 160 g/L glucose and supplemented with different concentrations of acetic acid.

Acetic Acid Concentration (g/L)	*P. kudriavzevii* CM4.2				*P. kudriavzevii* PkAC-9			
	*P* (g/L)	*Qp* (g/L·h)	*Yp/s* (g/g)	T (h)	*P* (g/L)	*Qp* (g/L·h)	*Yp*/*s* (g/g)	T (h)
0 (control)	54.60 ± 0.01 ^b^	3.03 ± 0.01 ^a^	0.46 ± 0.01 ^a^	18	58.35 ± 0.01 ^d^	3.24 ± 0.00 ^a^	0.49 ± 0.01 ^a^	18
7	54.82 ± 0.02 ^a^	0.65 ± 0.00 ^b^	0.45 ± 0.01 ^a^	84	63.67 ± 0.00 ^c^	0.76 ± 0.02 ^b^	0.50 ± 0.02 ^a^	84
8	47.65 ± 0.00 ^c^	0.40 ± 0.00 ^c^	0.38 ± 0.00 ^b^	120	65.98 ± 0.01 ^b^	0.55 ± 0.00 ^c^	0.50 ± 0.00 ^a^	120
9	21.92 ± 0.01 ^d^	0.16 ± 0.00 ^d^	0.16 ± 0.01 ^c^	132	66.25 ± 0.01 ^a^	0.50 ± 0.01 ^d^	0.50 ± 0.00 ^a^	132

*P*, ethanol concentration (g/L); *Qp*, volumetric ethanol productivity (g/L·h); *Yp*/*s*, ethanol yield (g/g); T, fermentation time that yielded the highest ethanol concentration. Means followed by different letters within the same column were statistically different at *p* ≤ 0.05 based on DMRT analysis.

## Data Availability

The experimental data are contained within the article.

## Supplementary Materials

The following supporting information can be downloaded at: https://www.mdpi.com/article/10.3390/jof11030177/s1. Table S1: Specific primers used to determine gene expression using RT-qPCR in this study.
