# Supplementary material for: Transcriptional Regulation Mechanisms in Adaptively Evolved Pichia kudriavzevii Under Acetic Acid Stress
Source: J Fungi (Basel). 2025 Feb 22;11(3):177. doi: 10.3390/jof11030177 (PMC11942776; doi:10.3390/jof11030177)
Supplement: Supplementary file 1 [file jof-11-00177-s001.zip › jof-3489142-supplementary.pdf]

Journal of *Fungi*

Article

Transcriptional Regulation Mechanisms in Adaptively Evolved *Pichia kudriavzevii* Under Acetic Acid Stress

Sureeporn Dolpatcha [1], Huynh Xuan Phong [2], Sudarat Thanonkeo [3], Preekamol Klanrit [1,4], Nongluck Boonchot [1,4], Mamoru Yamada [5,6] and Pornthap Thanonkeo [1,4,*]

1 Department of Biotechnology, Faculty of Technology, Khon Kaen University, Khon Kaen 40002, Thailand; sureeporndo@kkumail.com (S.D.); kpreek@kku.ac.th (P.K.); nongke@kku.ac.th (N.B.)
2 Department of Microbial Biotechnology, Institute of Food and Biotechnology, Can Tho University, Can Tho 900000, Vietnam; hxphong@ctu.edu.vn
3 Walai Rukhavej Botanical Research Institute, Mahasarakham University, Maha Sarakham 44150, Thailand; sudarat.t@msu.ac.th
4 Fermentation Research Center for Value Added Agricultural Products (FerVAAPs), Khon Kaen University, Khon Kaen 40002, Thailand
5 Department of Biological Chemistry, Faculty of Agriculture, Yamaguchi University, Yamaguchi 753-8515, Japan; m-yamada@yamaguchi-u.ac.jp
6 Research Center for Thermotolerant Microbial Resources, Yamaguchi University, Yamaguchi 753-8515, Japan
* Correspondence: portha@kku.ac.th; Tel.: +66-819743340

Table S1. Specific primers used to determine gene expression using RT-qPCR in this study.

No.	Primer name	Gene name	Sequences (5′ → 3′)
1	Hkr1–F	Signaling mucin HKR1	AGCTCAAGCAGCGAGACATT
	Hkr1–R		AGGTGCAGATTGTTGGGTGT
2	Opy2–F	Protein OPY2	CAAGTGCCAGACAAACGAGC
	Opy2–R		CCTGCAATCCCTCCAACCTT
3	Cdc24–F	Cell division control protein 24	AACACAAGTGTCTCCTCGCC
	Cdc24–R		GCCTGGGATTTTTGCAAGGG
4	Ste20–F	p21-activated kinase 1	GACGACTGCTACAATCGGGA
	Ste20–R		TCACTTGGACCCAACCACTT
5	Cla4–F	Serine/threonine protein kinase CLA4	GACGACTGCTACAATCGGGA
	Cla4–R		TCACTTGGACCCAACCACTT
6	Ste11–F	Mitogen-activated protein kinase kinase	CCCAGCAGCTAGTAACCCTG
	Ste11–R	kinase	TGACTGATGAGAGGAGTCGT
7	Ste50–F	Protein STE50	ACATAACGGGGCTGACCTTG
	Ste50–R		ATACCTCGCCTAGCGCATTC
8	Pbs2–F	Mitogen-activated protein	AACTTGTCCAGGTCGTCGTC
	Pbs2–R	kinase kinase	TGCTGTTTCCGTTTTGCCTG
9	Hog1–F	Mitogen-activated protein	ATTGGAATGGGCGCATTTGG
	Hog1–R	kinase HOG1	ACACCGGAGTGGAAAAAGGT
10	Sln1–F	Sensor histidine kinase SLN1	TCGTTGCCGAGTTGAAGAGT
	Sln1–R		GTTGAACGCTCTGTAACGCC
11	Ypd1–F	Phosphorylase intermediate protein YPD1	ATTGCTGATGGACGAGGACG
	Ypd1–R		GTCCCCATTGTTGTCGCCTA
12	Ssk1–F	Mitogen-activated protein kinase kinase	CCGTCTTCGACACCATCCAT
	Ssk1–R	kinase SSK1	GCGGGACAACAGGAGATGAA
13	Ssk2–F	Mitogen-activated protein kinase kinase	AGAGACGACATCGCTACGTG
	Ssk2–R	kinase SSK2	AAGAGGGGTGGTCATCTGGT

14	Ptp3–F<br>Ptp3–R	Tyrosine-protein phosphatase PTP3	ACCCCCTTTCCTCACAGTCT<br>TCTTAAGGTGATGGTCGTGGC
15	Hot1–F<br>Hot1–R	High-osmolarity-induced transcription protein 1	TGGGGAAACCGAATCTGGTG<br>CATTGGCATCATTGGCGTCG
16	Smp1–F<br>Smp1–R	Transcription factor SMP1	GAAGGCACATGAGTTGTCCA<br>CTTCTCGGTGTCATTCGACG
17	Gpd1–F<br>Gpd1–R	NAD+-dependent glycerol-3-phosphate dehydrogenase	GGTGTCCCCTGCTGAAAGAT<br>TTACCGGAACCAACAACCGT
18	Haa1–F<br>Haa1–R	Transcriptional activator	AAGTATGCCTGTGAGAGGTGC<br>CAGAAGTCACACTGGGTGGAA
19	Ppr1–F<br>Ppr1–R	Transcriptional activator	CACTGGCCGTGAGATACCAA<br>ACGTTTCCACGCTTTCTCCA
20	Set1–F<br>Set1–R	Histone methyltransferase	CGCACAAGATAAAGGCAGGC<br>TGCTGGCCAGTATGATTTGGT
21	Mac1–F<br>Mac1–R	Copper ion-sensing transcription factor	TGGACGGGGAAAAGTATGCG<br>CCCCCTTGACCGAACCAATA
22	Azf1–F<br>Azf1–R	Transcription factor AZF1	AGATCCGCCTTGTTCGCTAC<br>CGTGTCCGAGACTATCGTGG
23	Stb5–F<br>Stb5–R	Transcription factor	GGAGGTATCAAAGGGACGCC<br>ACTGGGTTGTCGTTCATCACA
24	Rtg1–F<br>Rtg1–R	Mitochondrial retrograde pathway	ACTTGCCGGAGTCACAACTT<br>GACGTCCGTCTTTGGTTCCT
25	Rtg3–F<br>Rtg3–R	Mitochondrial retrograde pathway	GCCAAGACTCTCCTAGTGACG<br>TCGACTTGGATTACCCACGC
26	Ino1–F<br>Ino1–R	Transcriptional factor	AGGACACCCTTACCTCCAACT<br>GGTACCGTTGTTACCACCCA
27	Sfl1–F<br>Sfl1–R	Transcriptional factor	CTCCGTCCACAGTTACACCT<br>GGCTCCTCAAGCATGGCATA
28	Leu3–F<br>Leu3–R	Transcriptional factor	GTTCAAAGCTGAAAGCCCCG<br>AGCAAGGATGTGGTTGTCTCT
29	Dal80–F<br>Dal80–R	Transcriptional factor	GGACTCGCCCTCTATTTGGG<br>TCTGGATGTCCTGTTCCCCT
30	Cat8–F<br>Cat8–R	Acetic acid-induced programmed cell death	ATGAGGGTCTCTGTTGCGTG<br>GAACGCCTTTCGTGCAAGTT
31	Skn7–F<br>Skn7–R	Transcriptional factor	GGTGCAACAAAGCCAAGGAG<br>ACACGGTAAAACTTGCTTGGTG
32	Arg81–F<br>Arg81–R	Transcriptional factor	CACTTGTAGGCGGCGTAAGA<br>ACGGAGCTTCACGTCATACC
33	Nrg1–F<br>Nrg1–R	Transcriptional factor	ATACACAGCACAGTCGTCGG<br>AGGGAGGGCATGGATATCGT
34	Nth1–F<br>Nth1–R	Neutral trehalase protein	TGCATCCGCCAATAGAGGAG<br>TGGATGGATCTGTAGCTGCG
35	Adh2–F<br>Adh2–R	Alcohol dehydrogenase 2	GCAATCTATTCCAAAGCCGGT<br>CCAGTTGTTGCAGCTAAGGC
36	Adh3–F<br>Adh3–R	Alcohol dehydrogenase 3	CCAGAGAAGCAAATGGGTGT<br>CGGTATGGCAGACACCAGAG
37	Gsk3–F<br>Gsk3–R	Glycogen synthase kinase	CCAGTGGTATTACCCGTCCC<br>TTGAGGGCTATTTGGCTCGG
38	Tdh2–F<br>Tdh2–R	Glyceraldehyde-3-phosphate dehydrogenase	AACGGTTTCGGTAGAATCGG<br>ACTTACCGTGTGTGGAGTCA
39	Hsp40–F	Heat shock protein 40	TTTGGACGGGCATTTACCGA

	Hsp40–R		GTCGTACTTCTTCTGCGCCT
40	Hsp60–F	Heat shock protein 60	AGCAGACGCTGTTTCTGTCA
	Hsp60–R		GCTTGGCACCCAAGTTTTCA
41	Ssb1–F	Heat shock protein 70	AGGTACTTTCGCTGGTGCAA
	Ssb1–R		TCTGGGGTGAAAGCAACGAA
42	Gsh–F	Pyrimidodoazepine synthase	CCCAGAAGGATTTCCCGGTTT
	Gsh–R		CAGAACGGGTTCGTAGTCCT
43	Gst–F	Glutathione S-transferase	TCCCCAACTCCAAATGGCTT
	Gst–R		GCCCTTTTCGTCAACGTGAG
44	Erg2–F	Sterol C-8 isomerase	CTCGTACCGAACCCTCGTTT
	Erg2–R		CTGGGTGCTGTGAAAAATGGA
45	Tps1–F	Trehalose-6-phosphate synthase	ATCCTGAGAGTGGCAACACC
	Tps1–R		AATACCTGGCCATCCGAACC
46	Lre1–F	Heat shock response	CCCTCGTTAGCCGAACCAAT
	Lre1–R		GGGTGTTAGCGGATGTGGTA
47	Sgt2–F	Heat shock response	AGCTTTCGCCGTTGAGTCTT
	Sgt2–R		CGTCGGACTCGTCAACATGA
48	Rrt12–F	Membrane biogenesis	GCTGCCTCCTTTTATTCACCTG
	Rrt12–R		TCACAGAGTCATCCTGCAAACA
49	Gas4–F	Membrane biogenesis	GAAATGAGCCATGGACCACA
	Gas4–R		CGGCCTTAATGTAAATCGGCG
50	Flo1–F	Membrane biogenesis	ACAGCACCCGAATCTGGTAT
	Flo1–R		ACTGCCAGGTGTTAGGTTGC
51	Hsp104–F	Heat shock protein 104	GTCCAGTGACGTTGGACGAA
	Hsp104–R		GCATCCCGCTGATGATGAGA
52	Ubp16–F	Ubiquitin-proteasome	TGGTTCCAATACGTTCTCCGA
	Ubp16–R		CCCGCTGTTTTTGAACCCAC
53	Bul2–F	Ubiquitin-proteasome	ACCATCAACGACAACGCAGA
	Bul2–R		ACTGAACTCACGTCATCGGT
54	Tom1–F	Ubiquitin-proteasome	GATCAGAGCCTGGTCCAGTT
	Tom1–R		GCTTCTTATTTGGTGATGGGCT
55	Bre1–F	Ubiquitin-proteasome	GTCCCTCAAACGTCCACCAT
	Bre1–R		ATAGAGGCCCGTCACTGGAA
56	Cue2–F	Ubiquitin-proteasome	CGCAAATTCAGCAGACCCTG
	Cue2–R		TCGTAACTTGACTGATCCCCC
57	Nadh–de–F	NADH dehydrogenase (ubiquinone)	GACTTGTGTCCGTCGCATCT
	Nadh–de–R	activity	TGCAATTCTGCAGTCTGCTC
58	Sdh–F	Succinate dehydrogenase activity-electron	CACCGATGCAGAGGAACAGT
	Sdh–R	transport	ACACCCGCTGCATAAGAGAC
59	CyC–R–F	Ubiquinol-cytochrome-C reductase	TGCGAGTTACAGGTGAGTGG
	CyC–R–R	activity	TGCGATGATCGAAATGCTTTGT
60	CyC–O–F	Mitochondrial cytochrome C oxidase	TGCAAAAACGGTTTGCCAGT
	CyC–O–R	assembly	GGAGCCAAGCATAATAAGCCG
61	ATPase–F	Proton transporting ATPase activity	CCCACCATTTGATTGCAGGC
	ATPase–R		AACGTTGGAACTGGTTGACA
62	Phc–F	Phosphoenolpyruvate carboxykinase	AGGACTGAATCCAGACGCTA
	Phc–R		TGCACCAGAAGAGGAAATGGT

63	Pdc–F<br>Pdc–R	Pyruvate decarboxylase	AGGTCAGCCCATGAATTGTCA<br>ACCGATTGCATAAGCCTCGT
64	Mad–F<br>Mad–R	Malate dehydrogenase	AAGTGACCGTTCCTCGTTCC<br>GCCCTTGTTGAAAAGCGGAG
65	Csy–F<br>Csy–R	Citrate synthase	TCGCAAGATCCGCAACTAGA<br>ACGGTCTTACCGTGCTTTGA
66	Aco–F<br>Aco–R	Aconitate hydratase	AGCAAGATCTGTCGCAAGAGT<br>AGGTGTGGTCTTCATGGTTGT
67	Fum–F<br>Fum–R	Fumarate hydratase	GGTGACATCGAAGTCCCAAA<br>AGCACCAGACTCAGCATTGA
68	Iso–F<br>Iso–R	Isocitrate dehydrogenase	CGCCTTGGAGAAAATCCACG<br>GACGCGTCTCTGGATTGGAT
69	SucCoA–F<br>SucCoA–R	Succinyl-CoA synthetase	GGTTCACCGGTAGACAAGCA<br>CAACAGTGGCAAAGACAGGC
70	Oxo–F<br>Oxo–R	2-oxoglutarate dehydrogenase	CCAAACAGTTCCCAAAGCACT<br>TGAACGGACTTAGGGTCTTGT
